# Treatment with Sildenafil Promotes Angiogenesis and Modulates Immune Response in Ischemic Muscle Tissue

**DOI:** 10.3390/cimb48030283

**Published:** 2026-03-06

**Authors:** Amelie Kuhs, Lisa Bobrowski, Katharina Elbs, Matthias Kübler, Philipp Götz, Christoph Arnholdt, Manuel Lasch, Elisabeth Deindl

**Affiliations:** 1 Walter-Brendel-Centre of Experimental Medicine, University Hospital, Ludwig-Maximilians-Universität München, 81377 Munich, Germany; amelie.kuhs@campus.lmu.de (A.K.); lisa.bobrowski@med.uni-muenchen.de (L.B.); katharina.elbs@med.uni-muenchen.de (K.E.); matthias.kuebler@med.uni-muenchen.de (M.K.); p.goetz@med.uni-muenchen.de (P.G.); christophjohannes.arnholdt@med.uni-heidelberg.de (C.A.); manuel.lasch@med.uni-muenchen.de (M.L.); 2 Biomedical Center, Institute of Cardiovascular Physiology and Pathophysiology, Ludwig-Maximilians-Universität München, 82152 Planegg-Martinsried, Germany; 3 Department of Cardiovascular Diseases, TUM University Hospital German Heart Center, 80636 Munich, Germany; 4 Center for Cardiovascular Research (DZHK), Munich Heart Alliance (MHA), Partner Site Munich, 81377 Munich, Germany; 5 Deutsches Zentrum Immuntherapie (DZI) and Comprehensive Cancer Center Erlangen-EMN (CCC ER-EMN), Friedrich-Alexander-Universität Erlangen-Nürnberg (FAU), 91054 Erlangen, Germany; 6 Department of Oral- and Cranio-Maxillofacial Surgery, Friedrich-Alexander-Universität Erlangen-Nürnberg (FAU), 91054 Erlangen, Germany; 7 Department of Otorhinolaryngology, Heidelberg University, 69120 Heidelberg, Germany; 8 Department of Ophthalmology, Heidelberg University, 69120 Heidelberg, Germany; 9 Department of Otorhinolaryngology, Head and Neck Surgery, Ludwig-Maximilians-Universität München, 81377 Munich, Germany

**Keywords:** sildenafil, PDE5 inhibitor, nitric oxide signaling, CGMP pathway, angiogenesis, ischemia, cardiovascular occlusive disease, endothelial cell proliferation, macrophage polarization, peripheral artery disease

## Abstract

Sildenafil, a selective phosphodiesterase-5 (PDE5) inhibitor, supports vascular remodeling, but its effects on angiogenesis and regeneration of ischemic muscle tissue are not fully understood. We investigated the function of sildenafil by employing a murine hindlimb model of ischemia, in which ischemia and angiogenesis is induced by femoral artery ligation (FAL) in the lower leg of mice. Then, 7 days after FAL or sham operation, gastrocnemius muscles of sildenafil-treated and control mice were isolated and processed for histological and immunofluorescence analyses. Sildenafil treatment led to reduced apoptotic areas within the ischemic tissue (ascertained via TUNEL assay) and increased angiogenesis, evidenced by a higher capillary-to-muscle fiber ratio and an augmented number of proliferating capillary cells (CD31^+^/CD45^−^/BrdU^+^), compared to controls. We observed a decrease in the total count of leukocytes (CD45^+^) in sildenafil-treated mice. Regarding macrophage infiltration, we found a reduced total number of macrophages (CD68^+^), along with a shift in macrophage polarization toward the pro-angiogenic and anti-inflammatory M2-like phenotype (CD68^+^/MRC1^+^). In summary, we show that sildenafil treatment contributes to angiogenesis and the regeneration of ischemic muscle tissue, most likely by attenuating inflammatory responses and influencing macrophage polarization in direction to regenerative M2-like polarized macrophages.

## 1. Introduction

Peripheral artery disease (PAD) is mainly caused by atherosclerosis and is associated with increased risk for cardiovascular complications and death. It is characterized by reduced arterial blood flow, leading to compromised blood supply to the extremities. This condition can lead to both acute and chronic circulatory problems in the lower leg, including intermittent claudication (IC) and chronic limb-threatening ischemia (CLTI) [1]. Due to increasing exposure to atherosclerotic risk factors and an aging population, the global prevalence of PAD continues to rise [2]. CLTI, the most severe end-stage of PAD, is often correlated with limb loss and high mortality. Current therapeutic options for CLTI patients include timely revascularization, whether surgical or endovascular, as well as medical therapy, consisting of anti-lipid-, anti-hypertensive-, and anti-platelet-therapy, to improve cardiovascular health. However, in some patients, revascularization is not possible due to comorbidities, high perioperative risk, or anatomical conditions, making amputation necessary [3]. Given these limitations, complementary strategies are needed to improve local tissue oxygenation in ischemic limbs. Promoting angiogenesis is a promising strategy, as angiogenic activity is a fundamental component of the tissue repair process and ultimately leads to the formation of new microvascular networks in ischemic tissue [4,5].

Angiogenesis, the formation of new capillaries from pre-existing vasculature, is driven by proliferation, migration, and differentiation of endothelial cells [6]. It can proceed via two main mechanisms: sprouting angiogenesis and intussusceptive (splitting) angiogenesis. Sprouting angiogenesis involves the outgrowth of new vessels from existing ones, initially resulting in immature vascular structures that later undergo stabilization and maturation [6,7]. In contrast, intussusceptive angiogenesis generates new vessels through intraluminal remodeling, whereby an existing vessel is subdivided into two functional branches [8,9].

Angiogenesis is tightly regulated by a balance of pro-angiogenic factors, such as vascular endothelial growth factor (VEGF), nitric oxide (NO), matrix metalloproteinases (MMPs), and anti-angiogenic mediators like interferon-α and interleukin-4 (IL-4) [10]. This process is primarily induced by ischemia [11]. Under ischemic conditions, VEGF-A expression is strongly upregulated via hypoxia-inducible factor 1α (HIF-1α), driving endothelial proliferation, migration, and remodeling through activation of vascular endothelial growth factor receptor-2 (VEGFR-2) [12,13]. Increased vascular permeability promotes the extravasation of plasma proteins, which form a scaffold for migrating endothelial cells [6].

The formation of new microvessels enhances local circulation, facilitating the recruitment of immune and phagocytic cells to the sites of injury. Leukocytes play a crucial role in angiogenesis by coordinating inflammatory and repair processes in damaged tissue [14]. During inflammation, monocytes differentiate into macrophages, which maintain and modulate angiogenesis by secreting angiogenic growth factors such as fibroblast growth factor (FGF), platelet-derived growth factor (PDGF), transforming growth factor-β (TGF-β), and VEGF. Macrophages also guide endothelial tip cells during vascular branching and anastomosis and contribute to vascular maturation depending on their activation state. Pro-inflammatory (M1-like) polarized macrophages initiate sprouting, whereas anti-inflammatory (M2-like) polarized macrophages promote vessel stabilization [15,16]. Leukocytes remove necrotic and inflammatory debris, while the reestablished microvascular network ensures efficient drainage of the waste and metabolic by-products, thereby promoting tissue repair and functional recovery [17,18]. Ischemic damage also leads to endothelial dysfunction, enhanced oxidative stress, and inflammation activation, thereby aggravating tissue damage beyond the ischemic stage [19,20,21,22].

Platelets have been shown to contribute to angiogenesis by releasing proangiogenic mediators such as VEGF, basic fibroblast growth factor (bFGF), and platelet-derived endothelial growth factor (PD-ECGF), thereby modulating angiogenic processes and vascular growth [23,24,25]. Additionally, platelets store anti-angiogenic factors such as platelet factor 4 (PF4), enabling them to terminate angiogenesis [25,26,27]. However, platelet aggregation would impair reperfusion in ischemic tissue. Thus, a nitric oxide (NO)-mediated signaling cascade counteracts platelet aggregate formation: Activation of the NO – soluble guanylate cyclase – cyclic guanosine monophosphate (NO-sGC-cGMP) signaling pathway leads to the protein kinase G (PKG)-mediated phosphorylation of vasodilator-stimulated phosphoprotein (VASP), which inhibits glycoprotein IIb/IIIa (GPIIb/IIIa) activation, thereby preventing platelet aggregation [28,29]. Thus, NO plays a crucial role in the process of angiogenesis by inducing vasodilation and by limiting platelet aggregation. Moreover, it has been shown that NO affects angiogenesis by stimulating proliferation and migration of endothelial cells and enhancing VEGF-A-mediated signaling [29,30].

Sildenafil, a potent phosphodiesterase-5 (PDE5) inhibitor, is primarily used for the treatment of erectile dysfunction and pulmonary hypertension [31]. By inhibiting cGMP degradation, sildenafil increases its intracellular concentration in platelets, cardiomyocytes, skeletal muscle, and smooth muscle cells and thereby enhancing NO signaling [32,33,34]. Previous studies have shown proangiogenic and immunomodulatory effects of sildenafil regarding vascular density, tissue perfusion, and modulation of macrophage polarization in ischemic muscle tissue via PKG-dependent signaling pathways, even under conditions of impaired NO synthase activity [35,36]. In experimental models of hindlimb ischemia, sildenafil has also been shown to attenuate skeletal muscle apoptosis via a reduction in caspase-3 activation, decrease TNF-α expression, and reduce oxidative stress markers following ischemic injury [21,22]. Moreover, sildenafil prevented ischemic-induced endothelial dysfunction in humans through activation of ATP-sensitive potassium channels [20].

The present study aimed to elucidate the mechanisms of sildenafil-promoted angiogenesis in a murine hindlimb model of ischemia, with a particular focus on the role of macrophage polarization in mediating vascular remodeling and tissue regeneration. Accordingly, sildenafil was administered prior to ischemia induction to enable mechanistic analysis of PDE5-dependent signaling under controlled conditions.

## 2. Materials and Methods

### 2.1. Animals and Treatments

Experimental procedures and animal care were performed with approval from the Bavarian Animal Care and Use Committee (ethical approval code: ROB-55.2Vet-2532.Vet_02-17-99, approval date 8 December 2017) under German animal legislation guidelines. Healthy 129/SV mice were either purchased from Charles River Laboratory (Sulzfeld, Germany) or bred in-house. The animals were held in a 12 h dark-light cycle in standard laboratory housing conditions and received standard laboratory food and drinking water ad libitum.

To investigate the effect of sildenafil on angiogenesis, we randomly assigned male mice aged 8–12 weeks to either treatment with sildenafil or control treatment with standard drinking water without drug addition. Each group consisted of 5 mice (*n* = 5). The animals were monitored every day. Group assignment was performed by simple randomization based on cage allocation. Due to practical limitations, the outcome assessment was not blinded, but standardized protocols were used to limit potential bias. No animal had to be excluded from the analysis because of adverse effects.

Sildenafil (CAS. Nr.: 171599-83-0, Hycultec, Beutelsbach, Germany) was administered in a dose of 10 mg/kg body weight via the provided standard laboratory drinking water (dilution 66.7 µg/mL, assuming a drinking amount of 15 mL/100 g body weight per mouse) from one day prior to femoral artery ligation until tissue removal 7 days after the procedure. To examine the proliferation rate of vascular cells, the mice received a daily intraperitoneal injection of bromodeoxyuridine (BrdU), a proliferation marker (1.25 mg/day, Sigma-Aldrich, St. Louis, MO, USA), dissolved in 100 µL sterile phosphate-buffered saline (PBS, PAN Biotech, Aidenbach, Germany, pH 7.4), beginning from the time of surgical intervention.

### 2.2. Surgical Procedure and Tissue Processing

The minimally invasive surgery involved the ligation of the right femoral artery to induce ischemia and stimulate angiogenesis in the gastrocnemius muscle. Mice were anesthetized with a combination of fentanyl (0.05 mg/kg, CuraMED Pharma, Karlsruhe, Germany), midazolam (5.0 mg/kg, Ratiopharm GmbH, Ulm, Germany) and medetomidine (0.5 mg/kg, Pfister Pharma, Berlin, Germany). To obtain an internal control, a sham surgery was performed on the left femoral artery [37].

Tissue sampling was performed 7 days after femoral artery ligation (*n* = 5 per group) under anesthesia, as described above.

After sacrifice under anesthesia 7 days after femoral artery ligation, the hindlimbs were perfused with an adenosine buffer (Sigma-Aldrich, Taufkirchen, Germany), a combination of 1% adenosine, 5% bovine serum albumin (BSA, Sigma-Aldrich, Taufkirchen, Germany) dissolved in PBS, and fixed with 3% paraformaldehyde (PFA, Merck, Darmstadt, Germany) for cryoconservation via aortic catheterization. The gastrocnemius muscles from both hindlimbs were then harvested, stored overnight in a 30% sucrose solution, embedded in Tissue Tek^®^ (REF 4583, Sakura Finetek, Torrance, CA, USA) and then stored at −80 °C for further analyses.

### 2.3. Immunohistological Analysis

For immunohistological analyses, 8 µm thick slices of the cryopreserved tissue samples were cut. They were used for stainings of apoptotic cells, leukocytes, endothelial cells, and macrophages.

To detect apoptotic cells in the gastrocnemius muscle tissue, the ApopTag^®^ Plus Fluorescein in Situ Apoptosis Detection Kit (EMD Millipore Corp., Burlington, MA, USA) was used in accordance with the instructions given by the manufacturer.

A combined staining of leukocytes (anti-CD45 antibody), endothelial cells (anti-CD31 antibody) and proliferating cells (anti-BrdU antibody) was performed to analyze the number of proliferating endothelial cells, as well as the number of leukocytes. Therefore, BrdU-treated muscle tissue sections were incubated with prewarmed 1N HCL in a humidifying chamber at 37 °C for 30 min. Hereafter, the samples were permeabilized with 0.2% Triton X-100 (AppliChem GmbH, Darmstadt, Germany) soluted in 4% BSA PBS/0.1%Tween-20 (AppliChem GmbH), followed by a 1 h blocking step with 10% goat serum (Abcam, ab7481, Cambridge, UK) dissolved in 4% PBS/0.1% Tween-20/0.5% BSA at room temperature. The sections then were incubated with the primary antibody rat anti-BrdU (Abcam, ab6326) diluted 1:50 in blocking solution overnight at 4 °C. Afterwards, incubated with the secondary antibody goat-anti-rat Alexa Fluor^®^ 546 antibody (1:100 in PBS/0.1%Tween-20 (PBST), Thermo Fisher, Waltham, MA, USA, A11081) for 1 h at room temperature, followed by a second blocking step with PBS/0.1% Tween-20/4% BSA lasting 30 min at room temperature. To label and differentiate endothelial cells (CD31^+^/CD45^−^) from leukocytes (CD45^+^), a solution consisting of the Alexa Fluor^®^ 647 anti-mouse CD31 antibody (1:50 in PBST, BioLegend, San Diego, CA, USA, 102516) and the anti-CD45-Alexa Fluor^®^ 488 antibody (1:100 in PBST, Thermo Fisher, 11-0451-85) was applied to the sections and incubated for 2 h.

To evaluate the number and polarization of macrophages, the sections were fixed with 4% PFA for 10 min at room temperature, followed by 1 h of blocking with 10% goat serum. The samples then were incubated with a monoclonal rat antibody against mannose receptor C type 1 (anti-MRC1) antibody (1:200 in goat serum, Invitrogen, Waltham, MA, USA, MA5-16871) at 4 °C overnight. The goat-anti-rat Alexa Fluor^®^ 546 antibody (1:100 in PBST, Thermo Fisher, A11081) served as secondary antibody, incubated for 1 h at room temperature. Subsequently, tissue sections were blocked with PBS/0.1% Tween-20/4% BSA lasting 30 min at room temperature and incubation with anti-CD68-Alexa Fluor^®^ 488 antibody (1:200 in PBST, Abcam, ab201844) at 4 °C overnight.

Furthermore, all stains were supplemented with 4′,6-Diamidino-2-phenylindol (DAPI) (1:1000 in PBST, Thermo Fisher, 62248) to label nucleic DNA, finally preserved with Dako mounting medium (Dako, Agilent, Santa Clara, CA, USA) and stored with protection from light at 4 °C until imaging.

Defined ischemic areas (1.5 mm^2^) in stained cross-sections of the gastrocnemius muscles were analyzed. Regions of interest (ROIs) were selected according to predefined morphological criteria and applied consistently across all animals. For each muscle, 5 images were captured from gastrocnemius muscle cross-sections and analyzed by quantifying muscle fibers and cells. Imaging was performed with a Leica DM6 B epifluorescence microscope (Leica Microsystems, Wetzlar, Germany) using the 20× objective (630 µm × 475 µm). Acquisition settings (exposure time, gain, illumination intensity) were defined prior to the first measurement and kept constant for all samples. Image processing and analysis were performed using the open-source program ImageJ (version 2.3.0, Wayne Rasband, retired from National Institutes of Health, Bethesda, MD, USA). Threshold values for fluorescence intensity and counting criteria were predefined prior to image analysis in ImageJ. The same threshold settings were applied uniformly to all sections across experimental groups to ensure comparability and to minimize analysis bias. Multiple sections per muscle were evaluated to reduce sampling bias.

### 2.4. Statistical Analyses

GraphPad Prism 8 (version 9.5.1, GraphPad Software, La Jolla, CA, USA) was used to perform statistical analyses and graphical design. Data are represented as means ± standard error of the mean (SEM). Results were considered statistically significant at *p* ≤ 0.05. The statistical test used for each analysis is described in the corresponding figure legend.

The sample size was calculated a priori using GPower software (version 3.1.9.2) based on the expected effect sizes derived from preliminary data and previous studies using the same animal model.

## 3. Results

To examine the effects of sildenafil on ischemia-induced angiogenesis in ischemic muscle tissue, a well-established murine hindlimb ischemia model was used [38]. To stimulate angiogenesis, tissue ischemia was induced by ligation of the right femoral artery in sildenafil-treated mice and untreated control mice. Meanwhile, the left leg underwent a sham surgery, serving as an internal negative control. For immunohistological analyses, gastrocnemius muscles were harvested 7 days after the surgical procedure.

**Figure 1:**
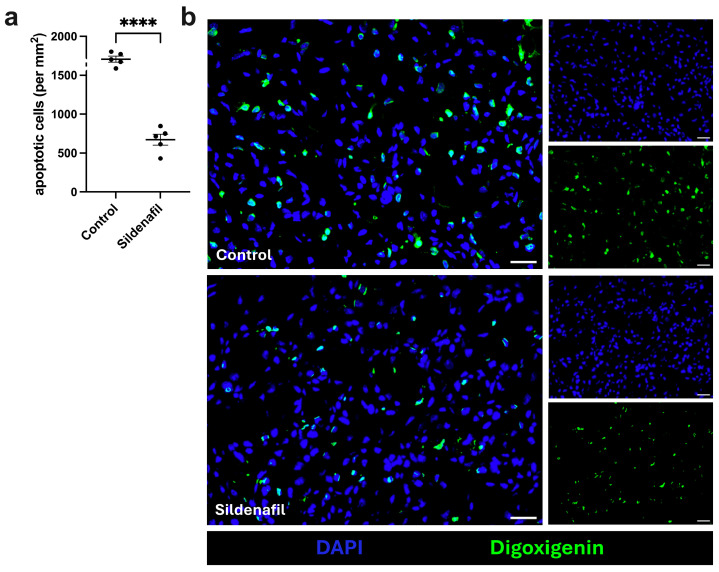
Treatment with sildenafil results in decreased numbers of apoptotic cells in ischemic muscle tissue. (**a**) The scatter plot displays the number of apoptotic cells per mm^2^ in control mice and sildenafil-treated mice 7 days after femoral artery ligation (aFAL). Analyses were performed on a defined ischemic muscle area (1.5 mm^2^) per mouse per group. The data presented are means ± SEM, with *n* = 5. **** *p* < 0.0001 determined by unpaired Student’s *t*-test. (**b**) Representative immunofluorescence images of TUNEL-stained ischemic gastrocnemius muscle from control mice (upper image) and sildenafil-treated mice (lower image) harvested 7 days aFAL. Cells were stained with DAPI to label nucleic DNA (blue) and with digoxigenin-labeled dUTP to detect DNA strand breaks (green) using the TUNEL (terminal deoxynucleotidyl transferase dUTP nick end labeling) assay. The larger images display the merged version of the smaller single channel images. Scale bars: 25 µm.

First, a terminal deoxynucleotidyl transferase dUTP nick-end labeling (TUNEL) assay was performed to assess the extent of ischemic tissue damage. Compared to control mice, sildenafil-treated mice showed a significantly reduced number of apoptotic cells (per mm^2^) at 7 days after FAL (Figure 1a,b; Figure S1). The sham-operated gastrocnemius muscle showed no signs of ischemic tissue damage (Figure S2).

**Figure 2:**
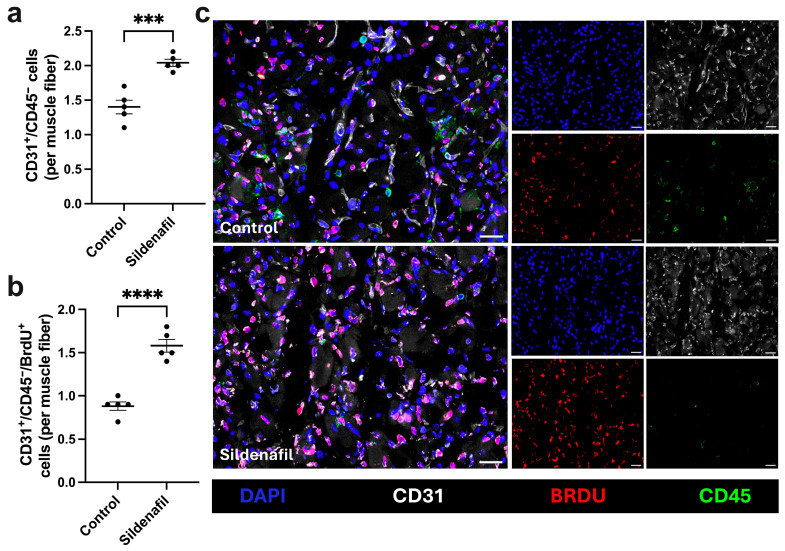
Capillarity is increasedin sildenafil-treated mice compared to control mice under ischemic conditions. The scatter plots illustrate (**a**) the number of endothelial cell (CD31^+^/CD45^−^) per muscle fiber and (**b**) the number of proliferating endothelial cells (CD31^+^/CD45^−^/BrdU^+^) per muscle fiber in control and sildenafil-treated mice. Analyses were conducted on ischemic gastrocnemius muscle tissue isolated 7 days after femoral artery ligation (aFAL). Analyses were performed on a defined ischemic muscle area (1.5 mm^2^) per mouse per group. The data shown are means ± SEM, with *n* = 5. *** *p* < 0.001, **** *p* < 0.0001 determined by unpaired Student’s *t*-test. (**c**) Representative images of ischemic gastrocnemius muscles of control (top) and sildenafil-treated mice (bottom) harvested 7 days aFAL. Single channel images show cell nuclei (DAPI, blue), endothelial cells (CD31, gray), proliferating cells (BrdU, red) and leukocytes (CD45, green). The larger images are a merged version of the single-channel images. Scale bars: 25 µm.

To determine whether sildenafil modulates angiogenesis, we performed quadruple immunofluorescence staining including CD31, CD45, BrdU, and DAPI on gastrocnemius muscle sections collected 7 days after FAL. The anti-CD31 antibody was implemented to identify endothelial cells, the anti-CD45 antibody to label leukocytes, the anti-BrdU antibody to mark proliferating cells, and DAPI to visualize cell nuclei. Since leukocytes are able to express CD31, only CD31^+^/CD45^−^ cells were quantified as endothelial cells. To analyze angiogenesis, the capillary-to-muscle fiber ratio, with muscle fibers identified by their autofluorescence, was calculated. Sildenafil-treated mice showed a significant increase in capillary-to-muscle fiber ratio compared to control mice (Figure 2a,c). Furthermore, sildenafil-treated mice exhibited significantly higher numbers of proliferating (BrdU^+^) endothelial cells per muscle fiber compared to controls (Figure 2b,c). No difference in the capillary-to-muscle fiber ratio was observed between control and sildenafil-treated mice in non-ischemic muscle tissue (Figure S3). No proliferation of endothelial cells was observed in sham operated muscle tissue from both experimental groups (Figure S3).

**Figure 3:**
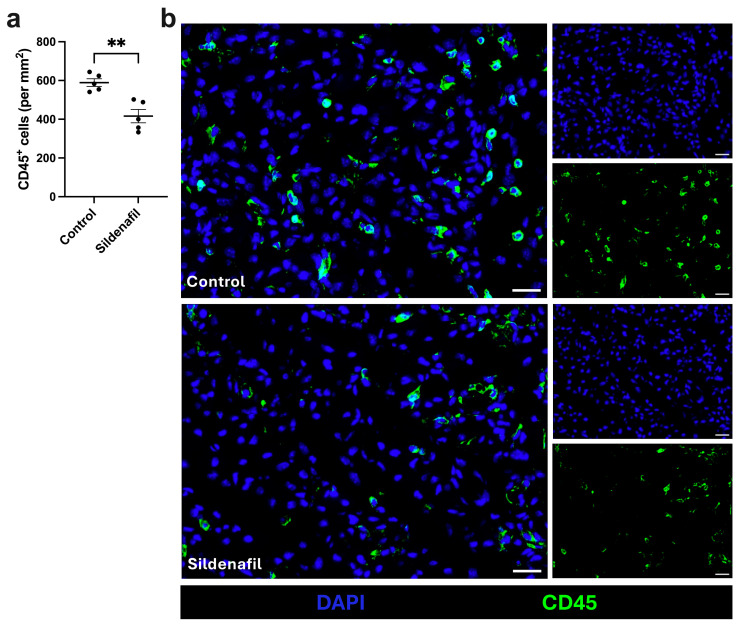
Sildenafil-treated mice show reduced leukocyte accumulation compared to control mice in ischemic muscle tissue. (**a**) Scatter plot displays the number of leukocytes (CD45^+^) per mm^2^ in ischemic gastrocnemius muscle tissue collected 7 days after femoral artery ligation (aFAL). A defined ischemic area (1.5 mm^2^) of ischemic gastrocnemius muscle tissue was analyzed per mouse. The data presented are means ± SEM, with *n* = 5 per group. ** *p* < 0.01 (control vs. sildenafil) determined by unpaired Student’s *t*-test. (**b**) Representative immunofluorescence images of gastrocnemius muscle from control (top) and sildenafil-treated mice (bottom) harvested 7 days aFAL. Leukocytes were labeled with an anti-CD45 antibody (green) and nuclei were stained using DAPI (blue). Scale bars: 25 µm.

As recruitment of inflammatory immune cells is known to support angiogenesis and tissue repair [39], we analyzed leukocyte accumulation in sildenafil-treated and control animals 7 days after FAL. To determine the number of leukocytes infiltrating the ischemic muscle tissue, we used the pan-leukocyte marker CD45. Here, we found a significantly lower number of leukocytes per mm^2^ in mice treated with sildenafil compared to control mice (Figure 3a,b). In the non-ischemic gastrocnemius muscle tissue, no significant difference in leukocyte counts between sildenafil-treated or control animals was detected (Figure S4).

**Figure 4:**
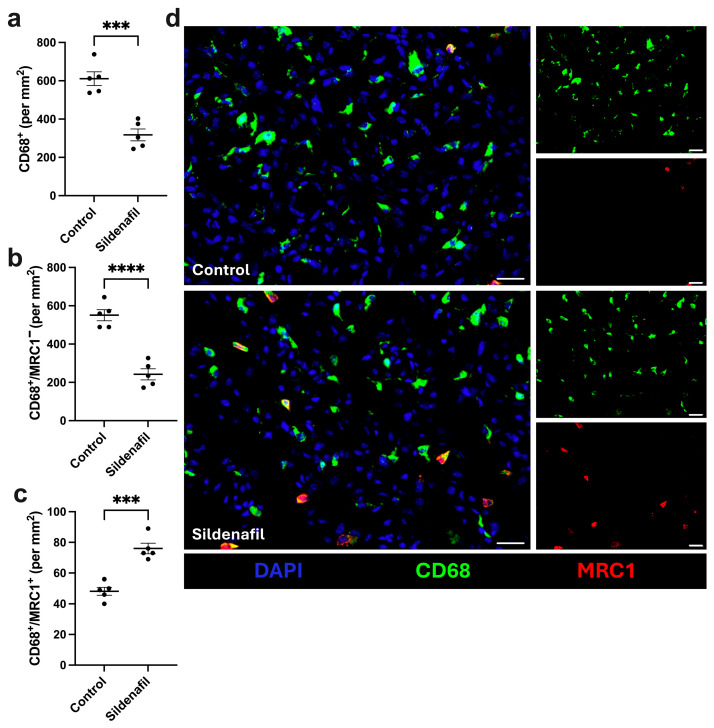
Sildenafil treatment reduces macrophage infiltration and favors polarization towards M2-like polarized macrophages. The scatter plots show (**a**) the number of macrophages (CD68^+^), (**b**) the number of M1-like polarized macrophages (CD68^+^/MRC1^−^), and (**c**) the number of M2-like polarized macrophages (CD68^+^/MRC1^+^) per mm^2^ in ischemic gastrocnemius muscles harvested 7 days after femoral artery ligation (aFAL). A defined ischemic area (1.5 mm^2^) of gastrocnemius muscle tissue was analyzed per mouse. Data presented are means ± SEM, with *n* = 5. *** *p* < 0.001, **** *p* < 0.0001 determined by unpaired Student’s *t*-test. (**d**) Representative images of ischemic gastrocnemius muscle of control (top) and sildenafil-treated mice (bottom). Antibodies targeting macrophages (CD68, green), M2-like polarized macrophages (MRC1, red) were used to label cells, while nuclei were counterstained with DAPI (blue). Scale bars 25 µm.

One crucial step in efficient angiogenesis is macrophage recruitment to ischemic sites, as their polarization determines the balance between pro- and anti-angiogenic signaling [15,40]. To investigate whether sildenafil-treatment influences macrophage infiltration and polarization in ischemic muscle tissue 7 days after FAL, we performed a staining using an anti-CD68 antibody to detect macrophages and an anti-mannose receptor C type 1 (MRC1) antibody to differentiate between inflammatory M1-like (MRC1^−^) polarized and regenerative M2-like (MRC1^+^) polarized macrophages. Our analyses revealed a significant decrease in the overall number of macrophages per mm^2^ in sildenafil-treated mice compared to controls (Figure 4a,d). Regarding macrophage polarization, ischemic muscle tissue sections from sildenafil-treated mice exhibited a significant decrease in pro-inflammatory M1-like polarized macrophages (CD68^+^/MRC1^−^) and an increase in anti-inflammatory M2-like polarized macrophages (CD68^+^/MRC1^+^) compared to controls (Figure 4b–d). In non-ischemic muscle tissue, no differences in macrophage infiltration or polarization were observed between sildenafil-treated or control animals (Figure S5).

## 4. Discussion

In the present study, we examined the influence of sildenafil on ischemia-induced angiogenesis and the accompanied leukocyte infiltration, with a particular focus on macrophage accumulation and polarization, using a well-established murine hind limb model of ischemia. Our data demonstrated that sildenafil treatment leads to reduced ischemic tissue damage along with an increased capillary-to-muscle fiber ratio, indicating enhanced angiogenesis. Furthermore, treatment with sildenafil results in reduced infiltration of immune cells (CD45^+^), in particular a decrease in the number of macrophages (CD68^+^). Interestingly, we observed a shift towards M2-like polarized regenerative macrophage polarization upon sildenafil treatment when compared to controls, marking its anti-inflammatory and regenerative impact on ischemia-induced angiogenesis.

Following femoral artery occlusion, downstream blood flow is reduced, leading to ischemia, local tissue damage, and fibrosis [5,13]. In order to maintain the metabolism in the ischemic area and to clear accumulated cell debris, angiogenic processes are initiated in parallel with the recruitment of local leukocytes [13]. Under hypoxic conditions, stabilization of the transcription factors HIF-1α and HIF-2α upregulates angiogenic mediators such as VEGF-A and stromal cell-derived factor 1α (SDF-1α), thereby enhancing angiogenesis [41,42]. The results of our TUNEL assay depict a significant reduction of ischemic tissue damage in sildenafil-treated mice compared to controls. This aligns with previous experimental studies demonstrating that treatment with sildenafil leads to upregulation of pro-angiogenic factors, thereby enhancing angiogenesis and promoting tissue repair and protection under ischemic conditions [43,44]. Notably, sildenafil has also been shown to exert direct anti-apoptotic effects in skeletal muscle following ischemia–reperfusion. In a rat hindlimb model, sildenafil administration significantly reduced caspase-3 activation and lowered the apoptotic ratio 24 h after reperfusion, supporting a myocyte-intrinsic anti-apoptotic mechanism [22]. In addition, reduced TNF-α immunoreactivity and decreased lipid peroxidation, reflected by lower malondialdehyde levels, have been reported in gastrocnemius muscle and femoral artery endothelium after sildenafil treatment in ischemia–reperfusion settings [21]. As previously demonstrated by Elbs et al., stimulation of the NO–cGMP signaling pathway with sildenafil significantly promotes collateral artery growth in the adductor muscle following FAL, resulting in increased collateral diameters and improved blood flow restoration [45]. Since ischemic damage in the gastrocnemius muscle critically depends on the efficiency of collateral artery development in the adductor region, the reduction in tissue damage observed in our study is therefore unlikely to be attributable solely to enhanced angiogenesis but may also reflect a substantial contribution from improved arteriogenesis.

Increased ischemic tissue damage reflects an intensified ischemic stimuli and thus is accompanied by increased angiogenesis [5]. Interestingly, enhanced angiogenesis occurred in sildenafil-treated mice compared to controls despite the overall reduction in ischemic tissue damage and, consequently, a lower expected angiogenic and inflammatory stimulus. This was confirmed by our immunohistological analyses, revealing a significant increase in endothelial cell proliferation and a higher ratio of capillaries-per-muscle-fiber in sildenafil-treated mice. Within the NO-GC/cGMP pathway, NO, mainly produced by endothelial nitric oxide synthase (eNOS), activates the sGC, which generates cyclic guanosine monophosphate. It has been shown that by preventing the degradation of cGMP, the PDE5-inhibitor sildenafil activates PKG-dependent signaling, which in turn stimulates the downstream PI3K/Akt and MAPK signaling cascades, and thereby promotes the survival, proliferation, and migration of endothelial cells [46,47,48,49,50].

In 2007, Pyriochou et al. demonstrated in both in vitro and in vivo studies that sildenafil induces endothelial tube formation and capillary sprouting via a PKG/MAPK-dependent mechanism [46]. In this context, our observations suggest that sildenafil promotes vascular growth through direct stimulation of endothelial signaling pathways, as increased angiogenesis was observed despite reduced ischemic damage, indicating that its pro-angiogenic effects are not solely driven by tissue ischemia.

Notably, Senthilkumar et al. highlighted sildenafil’s tissue-protective effects that occur independently of eNOS activity or NO production. This finding supports the assumption that pro-angiogenic and protective properties of sildenafil may involve additional cGMP-dependent mechanisms beyond classical NO-signaling, which would explain the simultaneous improvement in angiogenesis and reduction in ischemic tissue damage observed in our study [35].

In ischemic tissue, increased endothelial permeability leads to the infiltration of leukocytes, which remove cell debris, intensify local inflammation, and recruit additional immune cells, including neutrophils and macrophages [28,51]. Immune cells such as macrophages, dendritic cells, neutrophils, and T-cells also express PDE enzymes [52,53]. Sildenafil, as described, inhibits the catabolism of cGMP, leading to elevated intracellular cGMP levels [35]. High cGMP levels have been shown to reduce the production of pro-inflammatory cytokines, minimize oxidative stress, and downregulate the expression of endothelial adhesion molecules such as intracellular adhesion molecule-1 (ICAM-1) and vascular cell adhesion molecule-1 (VCAM-1) [54,55,56]. These cell adhesion molecules mediate leukocyte adherence to endothelial cells and facilitate leukocyte migration [57]. Moreover, cGMP not only affects leukocyte migration but also inflammatory signaling pathways by reducing proinflammatory factor release via the ERK1/2/NF-κB pathway [55,58,59,60].

In addition to the modulation of adhesion molecules and inflammatory signaling pathways, the endothelial glycocalyx may also represent mechanistic level contributing to the observed effects of sildenafil. The glycocalyx forms a dynamic interface between circulating blood components and the endothelial surface and plays a key role in regulating vascular permeability, leukocyte adhesion, platelet–endothelial interactions, and mechanotransduction [61,62,63,64]. Shear stress–mediated glycocalyx signaling promotes eNOS activation and NO production, whereas ischemia, oxidative stress, and inflammation induce glycocalyx shedding, thereby enhancing endothelial activation and immune cell recruitment [61,62,63,64]. Given that sildenafil enhances cGMP signaling and attenuates oxidative and inflammatory pathways, preservation of glycocalyx integrity may contribute to the reduced leukocyte infiltration and improved vascular remodeling observed in our study.

Beyond these molecular effects, sildenafil has been shown in a human in vivo model to prevent ischemia-induced endothelial dysfunction via the activation of ATP-sensitive potassium (KATP) channels [20]. This KATP-dependent endothelial protection may stabilize endothelial barrier integrity and reduce leukocyte adhesion, thereby contributing to the reduced inflammatory infiltration observed in our model.

Our study confirms these previous findings, demonstrating that sildenafil significantly reduces leukocyte infiltration, especially macrophage infiltration, in the ischemic tissue area. Furthermore, the vasodilatory effect of sildenafil may contribute to microvascular perfusion in ischemic areas, thereby attenuating hypoxia and reducing leukocyte infiltration [65,66]. Beyond its vascular effects, sildenafil also modulates inflammatory cell dynamics by influencing platelet activation and cytokine production [36].

Since excessive reactive oxygen species (ROS) generation and mitochondrial dysfunction are central drivers of ischemia induced skeletal muscle injury, the reduced leukocyte infiltration and attenuated tissue damage observed in our study may also reflect the indirect modulation of oxidative stress pathways. In experimental lower limb, mitochondrial maximal respiration and calcium retention capacity were markedly impaired, accompanied by increased ROS production [19]. Although sildenafil did not fully restore mitochondrial function in that acute setting, it tended to reduce ROS generation, suggesting partial redox modulation. Altogether, our study indicates that sildenafil reduces excessive inflammation and limits secondary tissue damage resulting from post-ischemic inflammatory processes.

Macrophages are known to be key regulators of both inflammation and angiogenesis. Their high plasticity allows them to exert opposing effects depending on local stimuli in ischemic tissues [51]. Classically activated M1-like polarized macrophages, induced by TNF-α, interferon-γ, and lipopolysaccharides (LPS), secrete pro-inflammatory cytokines such as IL-1β, IL-6 and TNF-α [15,17]. In contrast, M2-like polarized macrophages activated by IL-4 and IL-13, release not only anti-inflammatory but pro-angiogenic cytokines, including IL-10 and TGF-β [15]. Importantly, both M1- and M2-like polarized macrophages contribute to angiogenesis through the secretion of VEGF-A, at different stages: M1-like-polarized macrophages initiate capillary sprouting via the release of VEGF-A and fibroblast growth factor (FGF2), whereas M2-like polarized macrophages promote vessel maturation through VEGF-A, platelet-derived growth factor (PDGF-BB), and matrix metalloproteinase-9 (proMMP-9) [15,16]. In the context of ischemia, transient M1-like polarized macrophage activity supports early angiogenic sprouting by releasing VEGF, IL-8, and TNF-α. However, prolonged M1 accumulation can sustain inflammation and lead to vessel regression [67]. It is assumed that the timing of M1-like polarized macrophage activity is a critical factor for different outcomes [68]. Upon exposure to specific interleukins, M1-like polarized macrophages can repolarize into regenerative, anti-inflammatory M2-like polarized macrophages [69]. The M2-like polarized macrophages facilitate angiogenesis by downregulating tissue inhibitor of metalloproteinase 1 (TIMP-1), leading to the release of proMMP-9 and thereby facilitating extracellular matrix remodeling [70].

Several studies have examined the effect of sildenafil on macrophages. The results of these studies show that sildenafil, via an increase of CCR-2/MCP-1 associated with the cGMP/PKG pathway, promotes a switch from M1- to M2-like polarized macrophages. Furthermore, the expression of MMP-9 was increased after treatment with sildenafil [36,60]. Furthermore, it has been shown that sildenafil treatment leads to a reduction of TNF-α expression in femoral artery endothelium and skeletal muscle following ischemia, which may directly influence macrophage polarization dynamics [21]. Since TNF-α is a potent driver of M1-like polarization, its attenuation may facilitate the observed shift toward a regenerative M2-like phenotype in our study.

While sildenafil treatment was strongly associated with altered macrophage polarization in vivo, the observed effects likely reflect both direct immunomodulatory actions and secondary changes related to the improved tissue environment. Macrophage activation is highly plastic and critically shaped by the local tissue microenvironment [40,71]. Accordingly, reduced necrosis, hypoxia, and pro-inflammatory cytokine release may secondarily promote a regenerative phenotype [40,71]. At the same time, macrophages express phosphodiesterases that regulate intracellular cGMP signaling [36]. Inhibition of these enzymes has been shown to attenuate proinflammatory signaling pathways while promoting anti-inflammatory activation states [72,73]. A cell-intrinsic contribution of PDE5 inhibition is therefore biologically plausible. In our model, the polarization shift occurred in parallel with enhanced angiogenesis and reduced leukocyte infiltration, indicating coordinated immunovascular remodeling.

Collectively, our findings suggest that the enhanced angiogenesis observed following sildenafil treatment is associated with the promotion of an anti-inflammatory microenvironment, characterized by reduced leukocyte recruitment and a shift of macrophages toward a regenerative M2-like phenotype. This is the first study to demonstrate a link between sildenafil-induced macrophage polarization and angiogenesis in the context of ischemic tissue repair.

Beyond these direct immunomodulatory effects, current findings point to an additional level of regulation linking platelet activity to macrophage polarization and angiogenesis. Platelet-derived growth factor-D (PDGF-D) has been shown to activate both the classical (C1qa) and alternative (C3) complement pathways in retinal pigment epithelial cells, leading to the generation of complement fragments such as C3a and C5a. These complement-derived mediators induce the expression of chemokines and cytokines that attract and activate macrophages, promoting their polarization toward pro-angiogenic phenotypes characterized by enhanced VEGF, IL-10, and MMP-9 expression. Through this complement-dependent macrophage activation, PDGF-D drives inflammatory angiogenesis and vascular remodeling in the retina [74]. Similar platelet-mediated effects have been observed in osteoarthritis, where platelet-rich plasma promoted an anti-inflammatory M2-like polarized macrophage phenotype associated with increased IL-10 and TGF-β expression [75]. Since sildenafil modulates platelet function via the NO-sGC-cGMP signaling pathway, it is conceivable that part of its proangiogenic effect is based on indirect regulation of macrophage behavior through altered platelet activation.

In conclusion, we demonstrate that sildenafil treatment reduces the recruitment of leukocytes and is associated with a shift toward a regenerative M2 phenotype. This shift creates an anti-inflammatory environment, decreases tissue damage, and enhances angiogenesis. Therefore, sildenafil has significant immunomodulatory potential beyond its known vasodilatory effects.

These findings provide mechanistic insight into the role of NO–cGMP signaling in ischemia-induced vascular and immune responses. As peripheral artery disease is characterized by impaired microvascular adaptation to ischemia, modulation of this pathway may enhance endogenous angiogenic responses within ischemic tissue [76]. By promoting endothelial cell proliferation and supporting a regenerative macrophage phenotype, sildenafil may facilitate capillary remodeling in the peri-ischemic environment following surgical or endovascular revascularization. Based on these mechanisms, adjunctive perioperative sildenafil administration could potentially support microvascular recovery after procedures such as bypass grafting or endovascular intervention.

## Data Availability

The original contributions presented in this study are included in the article/Supplementary Materials. Further inquiries can be directed to the corresponding author.

## Supplementary Materials

The following supporting information can be downloaded at https://www.mdpi.com/article/10.3390/cimb48030283/s1.
