# Supplementary material for: Treatment with Sildenafil Promotes Angiogenesis and Modulates Immune Response in Ischemic Muscle Tissue
Source: Curr Issues Mol Biol. 2026 Mar 6;48(3):283. doi: 10.3390/cimb48030283 (PMC13024863; doi:10.3390/cimb48030283)
Supplement: Supplementary file 1 [file cimb-48-00283-s001.zip › cimb-4173789-supplementary.pdf]

Article

Treatment with Sildenafil Promotes Angiogenesis and Modulates Immune Response in Ischemic Muscle Tissue

Amelie Kuhs [1,2], Lisa Bobrowski [1,2], Katharina Elbs [1,2,3,4], Matthias Kübler, [1,2,5,6], Philipp Götz [1,2,7], Christoph Arnholdt [1,2,8], Manuel Lasch [1,2,9] and Elisabeth Deindl [1,2,*]

1 Walter-Brendel-Centre of Experimental Medicine, University Hospital, Ludwig-Maximilians-Universität München, 81377 Munich, Germany; amelie.kuhs@campus.lmu.de (A.K.); lisa.bobrowski@med.uni-muenchen.de (L.B.); katharina.elbs@med.uni-muenchen.de (K.E.); matthias.kuebler@med.uni-muenchen.de (M.K.); p.goetz@med.uni-muenchen.de (P.G.); christophjohannes.arnholdt@med.uni-heidelberg.de (C.A.); manuel.lasch@med.uni-muenchen.de (M.L.)

2 Biomedical Center, Institute of Cardiovascular Physiology and Pathophysiology, Ludwig-Maximilians-Universität München, 82152 Planegg-Martinsried, Germany

3 Department of Cardiovascular Diseases, TUM University Hospital German Heart Center, 80636 Munich, Germany

4 Center for Cardiovascular Research (DZHK), Munich Heart Alliance (MHA), Partner Site Munich, 81377 Munich, Germany

5 Deutsches Zentrum Immuntherapie (DZI) and Comprehensive Cancer Center Erlangen-EMN (CCC ER-EMN), Friedrich-Alexander-Universität Erlangen-Nürnberg (FAU), 91054 Erlangen, Germany

6 Department of Oral- and Cranio-Maxillofacial Surgery, Friedrich-Alexander-Universität Erlangen-Nürnberg (FAU), 91054 Erlangen, Germany

7 Department of Otorhinolaryngology, Heidelberg University, 69120 Heidelberg, Germany

8 Department of Ophthalmology, Heidelberg University, 69120 Heidelberg, Germany

9 Department of Otorhinolaryngology, Head and Neck Surgery, Ludwig-Maximilians-Universität München, 81377 Munich, Germany

* Correspondence: elisabeth.deindl@med.uni-muenchen.de; Tel.: +49-(0)-89-2180-76504

Supplementary Material

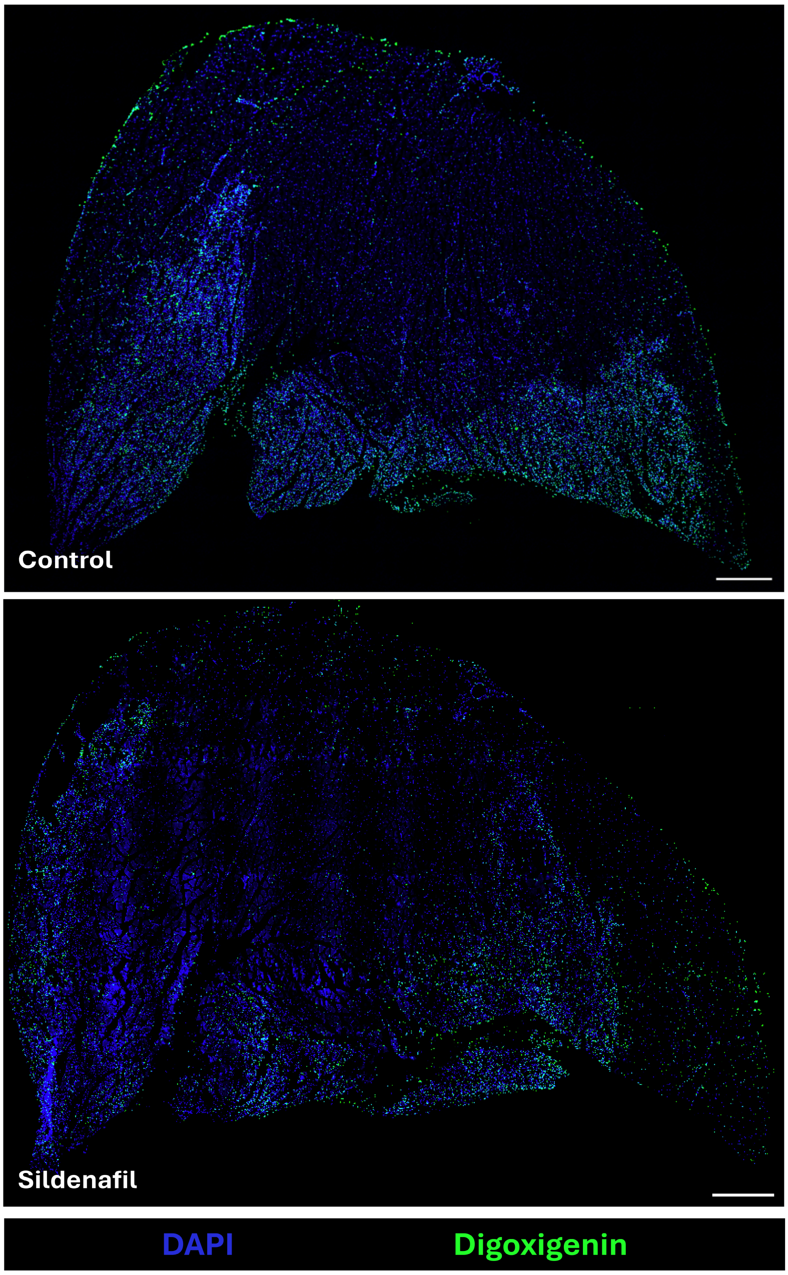

Figure S1. Representative overview of an entire ischemic gastrocnemius muscle cross-section following TUNEL-staining of control mice (upper image) and sildenafil-treated (lower image) mice 7 days aFAL. The image illustrates the overall staining pattern without quantitative evaluation, corresponding to the areas analyzed in Figure 1a, b of the main text. Scale bars: 500 µm.

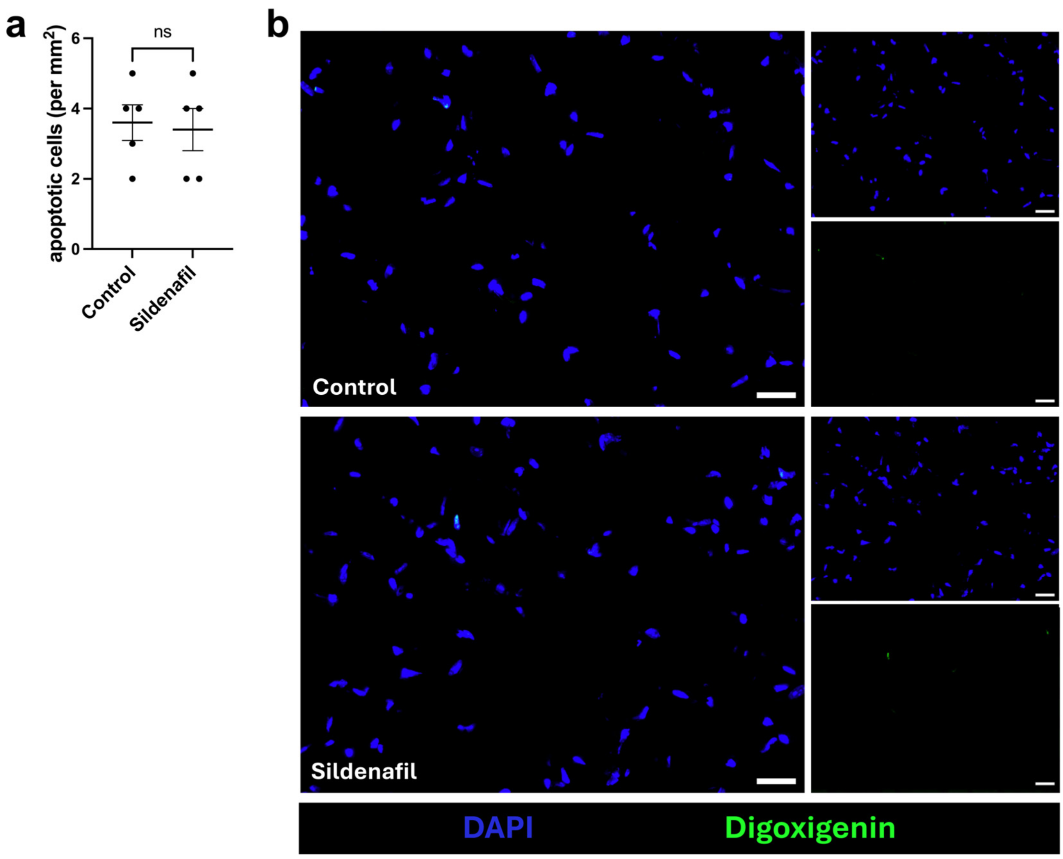

Figure S2. (a) The scatter plot shows no significant difference in the number of apoptotic cells per mm^2 in control mice and sildenafil-treated mice 7 days after sham operation. Analyses were performed on a defined muscle area (1,5 mm^2) per mouse per group. The data presented are means ± SEM, with n = 5. ns > 0.05 (control vs sildenafil) determined by unpaired Student´s t-test. (b) Representative immunofluorescence images of TUNEL-stained gastrocnemius muscle from sham control (upper image) and sildenafil-treated mice (lower image). Cells were stained with DAPI to visualize nuclear DNA (blue) and with digoxigenin-labeled dUTP to detect DNA strand breaks (green) via the TUNEL assay. The larger images display the merged version of the smaller single channel images. Scale bars 25µm.

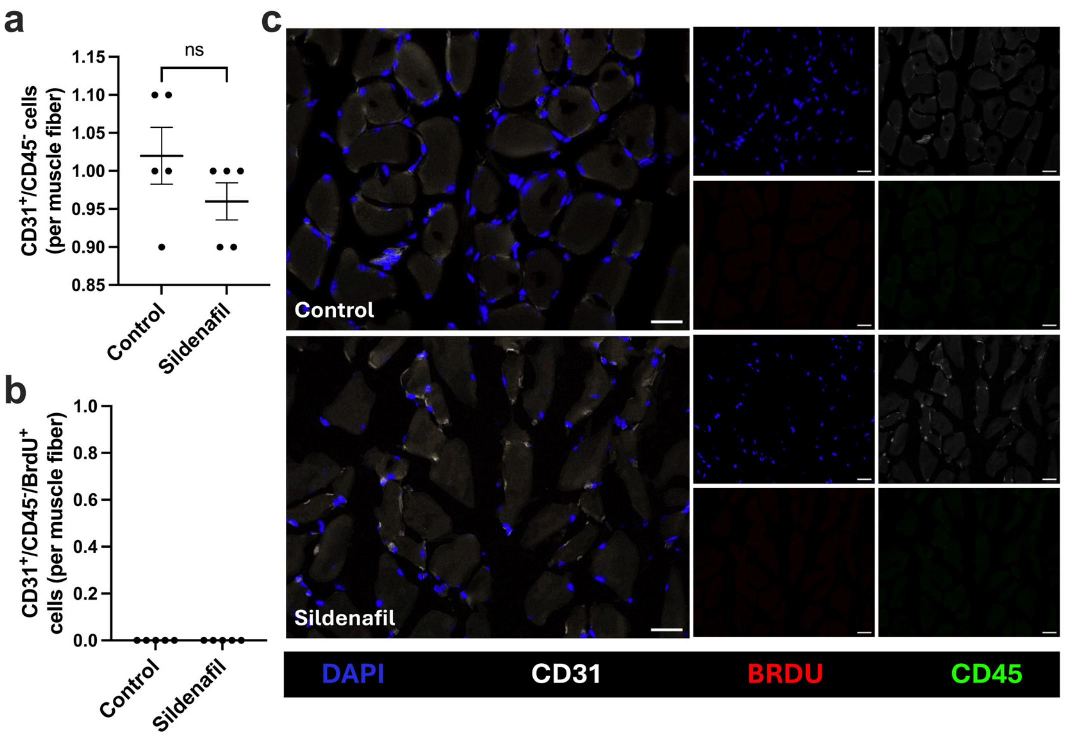

Figure S3. Sham-operated gastrocnemius muscle of control and sildenafil-treated mice displays no significant difference in capillarity and no proliferation of endothelial cells 7 days after sham operation. The scatter plots show (a) the number of endothelial cell ($CD31^+/CD45^-$) per muscle fiber and (b) the number of proliferating endothelial cells ($CD31^+/CD45^-/BrdU^+$) per muscle fiber in sham control and sildenafil-treated mice. Analyses were performed on a defined area (1,5 mm^2) of gastrocnemius muscle tissue harvested 7 days after operation. The data shown are means ± SEM, with n = 5. ns > 0.05 (control vs sildenafil) determined by unpaired Student´s t-test. (c) Representative images of gastrocnemius muscles of control (top) and sildenafil-treated mice (bottom). Antibodies directed against CD45 (leukocytes, green), CD31 (endothelial cells, grey), BrdU (proliferating cells, red) and DAPI (nuclei, blue) were used to mark cells. Scale bars 25µm.

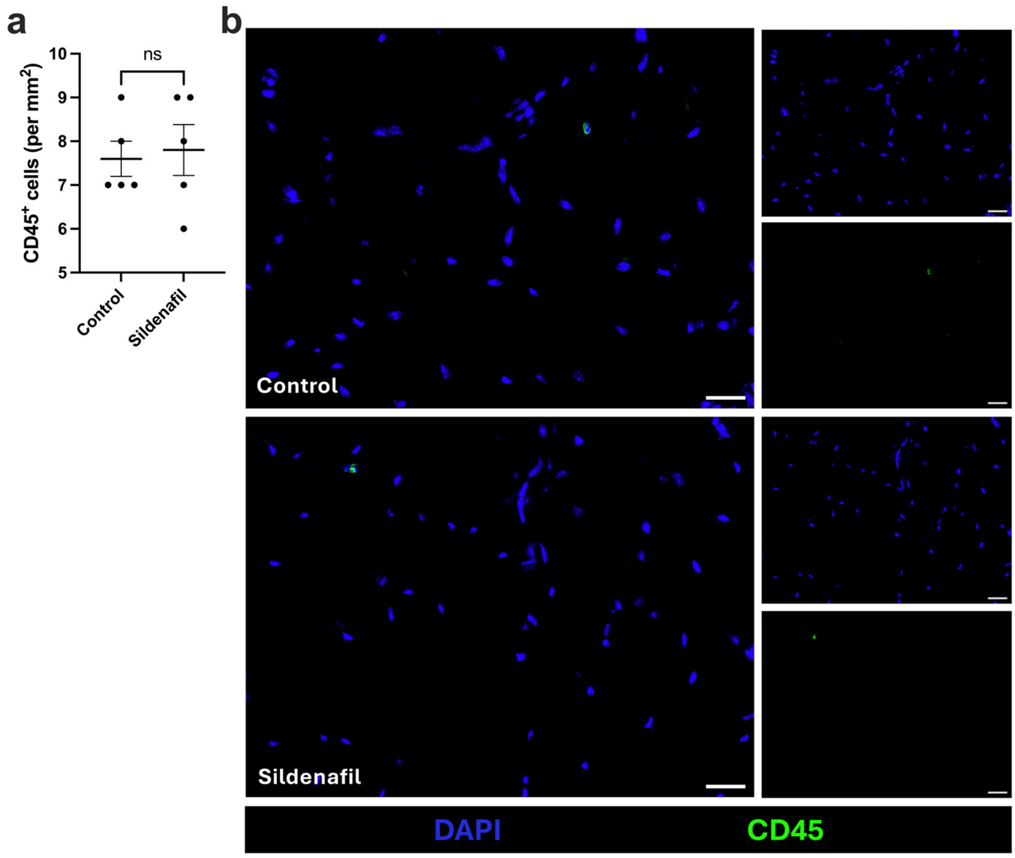

Figure S4. Representative pictures of control and sildenafil-treated mice show a low leukocyte count 7 days after sham operation. (a) Scatter plot illustrate no significant difference in the number of leukocytes ($CD45^+$) per mm^2. A defined area (1,5 mm^2) of gastrocnemius muscle tissue was analyzed per mouse. The data presented are means ± SEM, with n = 5 per group. ns > 0.05 (control vs sildenafil) determined by unpaired Student's t-test. (b) Representative immunofluorescence analyzed images of gastrocnemius muscle from sham control (upper image) and sildenafil-treated mice (lower image) at 7 days after sham operation. Leukocytes were labeled with CD45 (green), and nuclei were stained using DAPI (blue). Scale bars: 25 µm.

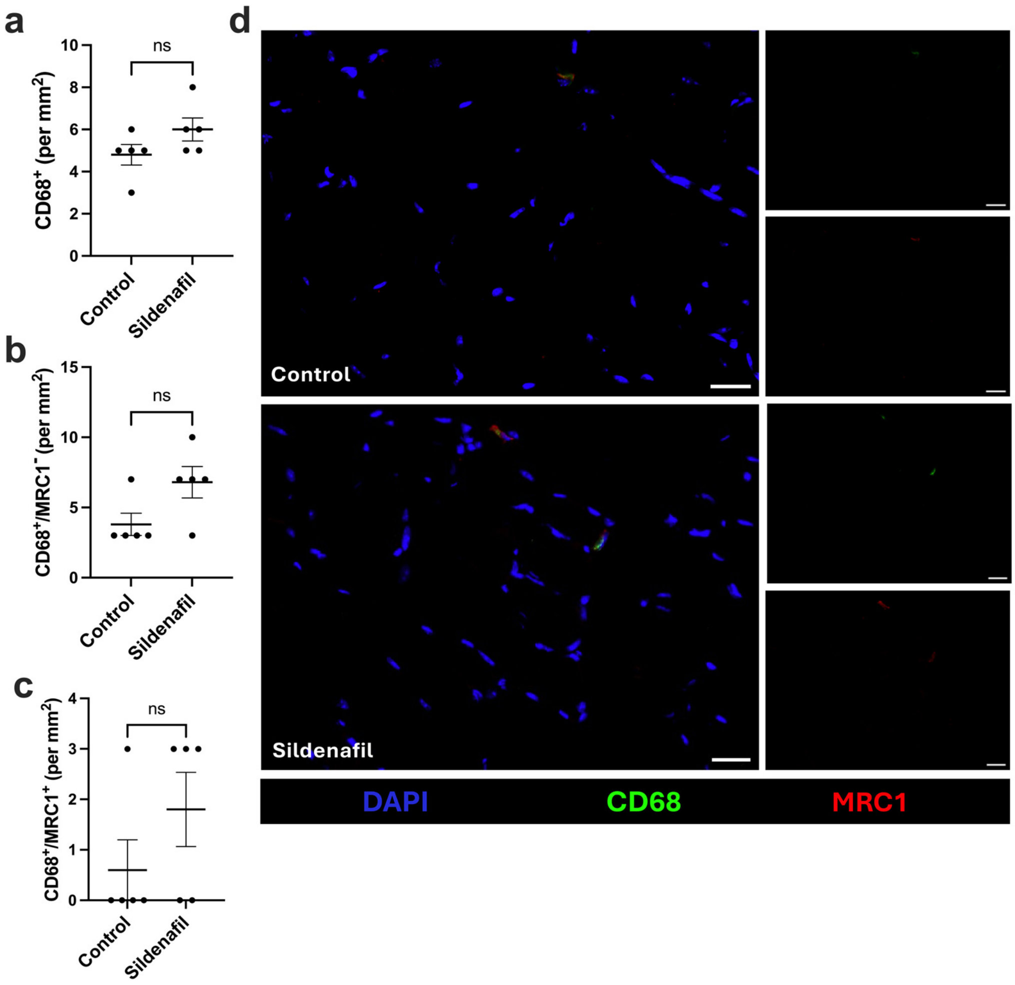

Figure S5. Sham-operated control and sildenafil-treated mice display low numbers of macrophages 7 days after surgical intervention. The scatter plot shows no significant difference in (a) the number of macrophages ($CD68^+$) per mm^2, (b) the number of M1-like polarized macrophages ($CD68^+/MRC1^-$), and (c) the number of M2-like polarized macrophages ($CD68^+/MRC1^+$). A defined area (1,5 mm^2) of gastrocnemius muscle tissue was analyzed per mouse. Data are shown as means ± SEM, with n=5. ns $p > 0.05$ (control vs sildenafil) determined by unpaired Student´s t-test. (d) Representative images of control (top) and sildenafil-treated mice (bottom). Cells were labelled with antibodies targeting CD68 (green, macrophages), MRC1 (red, M2-like polarized macrophages) and DAPI (blue, nuclei). Scale bars 25µm.
